# Determination of Baroreflex Sensitivity during the Modified Oxford Maneuver by Trigonometric Regressive Spectral Analysis

**DOI:** 10.1371/journal.pone.0018061

**Published:** 2011-03-18

**Authors:** Julia Gasch, Manja Reimann, Heinz Reichmann, Heinz Rüdiger, Tjalf Ziemssen

**Affiliations:** 1 Autonomic and Neuroendocrinological Laboratory, Department of Neurology, University Hospital Carl Gustav Carus, Dresden University of Technology, Dresden, Germany; 2 Research Group Neuro-Metabolism, Department of Neurology and Internal Medicine III, University Hospital Carl Gustav Carus, Dresden University of Technology, Dresden, Germany; University of Modena and Reggio Emilia, Italy

## Abstract

**Background:**

Differences in spontaneous and drug-induced baroreflex sensitivity (BRS) have been attributed to its different operating ranges. The current study attempted to compare BRS estimates during cardiovascular steady-state and pharmacologically stimulation using an innovative algorithm for dynamic determination of baroreflex gain.

**Methodology/Principal Findings:**

Forty-five volunteers underwent the modified Oxford maneuver in supine and 60° tilted position with blood pressure and heart rate being continuously recorded. Drug-induced BRS-estimates were calculated from data obtained by bolus injections of nitroprusside and phenylephrine. Spontaneous indices were derived from data obtained during rest (stationary) and under pharmacological stimulation (non-stationary) using the algorithm of trigonometric regressive spectral analysis (TRS). Spontaneous and drug-induced BRS values were significantly correlated and display directionally similar changes under different situations. Using the Bland-Altman method, systematic differences between spontaneous and drug-induced estimates were found and revealed that the discrepancy can be as large as the gain itself. Fixed bias was not evident with ordinary least products regression. The correlation and agreement between the estimates increased significantly when BRS was calculated by TRS in non-stationary mode during the drug injection period. TRS-BRS significantly increased during phenylephrine and decreased under nitroprusside.

**Conclusions/Significance:**

The TRS analysis provides a reliable, non-invasive assessment of human BRS not only under static steady state conditions, but also during pharmacological perturbation of the cardiovascular system.

## Introduction

The baroreflex circuit is crucial for maintenance of cardiovascular homeostasis. In consequence baroreflex dysfunction has been associated with a worse outcome in diabetes mellitus [1] and after myocardial infarction [2]. Intervention studies convincingly show that correction of baroreflex sensitivity (BRS) confers a reduced mortality risk [2], [3], [4]. Hence, BRS determination is increasingly used as prognostic tool. The pharmacological based modified Oxford method is considered to be the gold standard of BRS estimation but is often contraindicated in multimorbide patients [5], [6], [7]. Drug-induced effects have also been discussed to alter the measured parameter itself [5], [8], [9], [10], [11]. In recent years, non-invasive computer-assisted techniques have been established measuring BRS. There are clear-cut discrepancies between pharmacologically-induced and spontaneous BRS values which should not be interpreted as a difference between "real" and "biased" BRS estimates but rather as the expected difference resulting from methods that explore baroreflex function from different but complementary perspectives [12].

The EuroBaVar study compared 21 currently available methods of spontaneous BRS estimation [13] differing in their general approach (e.g. analysis in time vs. in frequency domain) and statistical algorithms (e.g. Fast Fourier Transformation vs. Autoregressive method). The Trigonometric Regressive Spectral (TRS) analysis technique showed an excellent performance as it dealt with two major problems: TRS detects real physiological oscillations rendering theoretical assumptions of the original data in a certain model system unnecessary. Problems like different interpolations of non-equidistant RR intervals, insufficient frequency resolution, aliasing, different lengths of the data segment needed, are minimised thereby enhancing statistical certainty of BRS estimation [14]. Given the profound variability of biological processes TRS uses very short time windows which are shifted beat by beat allowing for temporal resolution of frequency and amplitude [15], [16], [17], [18].

In the present study we attempted to compare BRS estimates derived from the modified Oxford method with those calculated by TRS during resting steady state and modified Oxford maneuver in the lying position and during head-up tilt-table test (HUT). For the first time we also aimed to analyse the dynamic modulation of BRS during the course of a modified Oxford maneuver.

## Results

We evaluated data of 45 healthy subjects comprising 22 men and 23 women with a mean age of 40±16 years. HUT evoked slight presyncopal symptoms in two volunteers in whom the trial was successfully repeated. Another volunteer experienced significant presyncopal symptoms resulting in discontinuation of the test.

**Figure 1:**
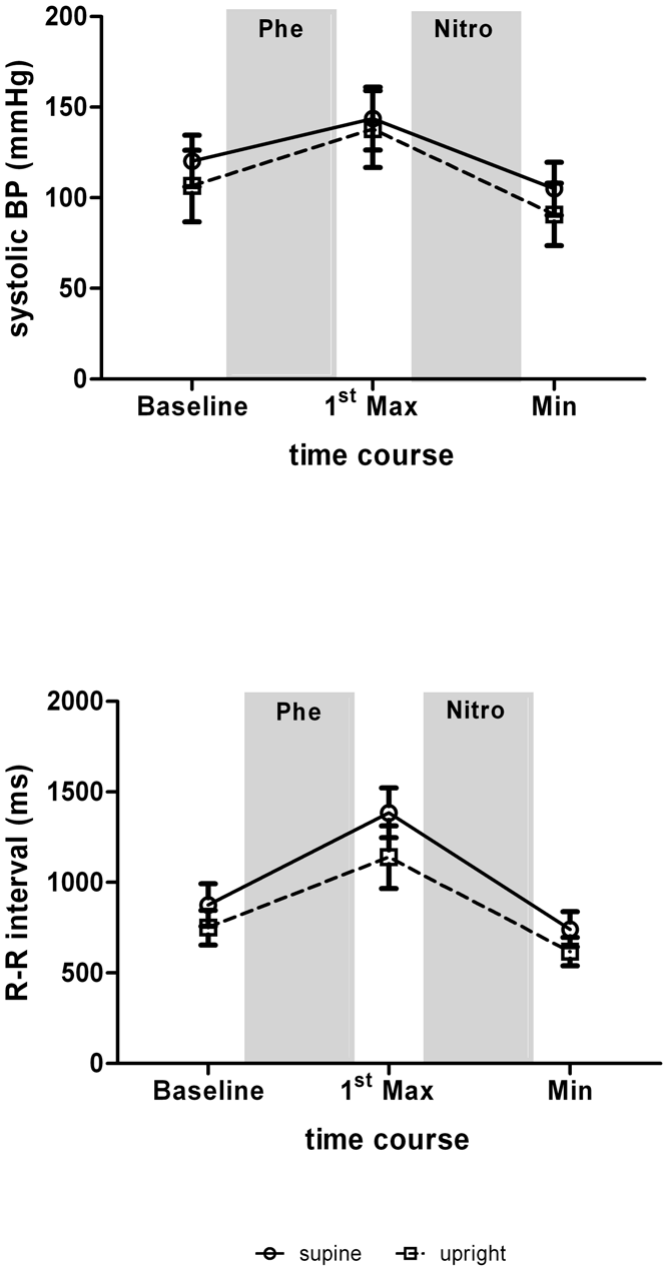
Systolic blood pressure (BP) and R-R interval (RRI) during the modified Oxford maneuver. Data are means ± SD.

The modulation of systolic blood pressure (SBP) and RR intervals (RRI) in response to pharmacological stimulation with phenylephrine and nitroprusside is illustrated in Figure 1. In the supine position, the SBP increased by 23.4±8.9 mmHg after phenylephrine (p_Δ_<0.001) and decreased by 38.0±14.0 mmHg after nitroprusside (p_Δ_<0.001). In the 60° upright position, an even stronger rise (31.2±9.7 mm Hg; p_Δ_<0.001) and fall (48.6±16.0 mmHg; p_Δ_<0.001) in SBP was observed. Inversely, RRI showed stronger increases (509.4±210.8 ms vs. 388.6±149.2 ms) and decreases (643.3±211.6 ms vs. 526.1±147.9 ms) during lying than standing (p_Δ_<0.001).

### Comparing spectrally and pharmacologically determined BRS estimates

**Figure 2:**
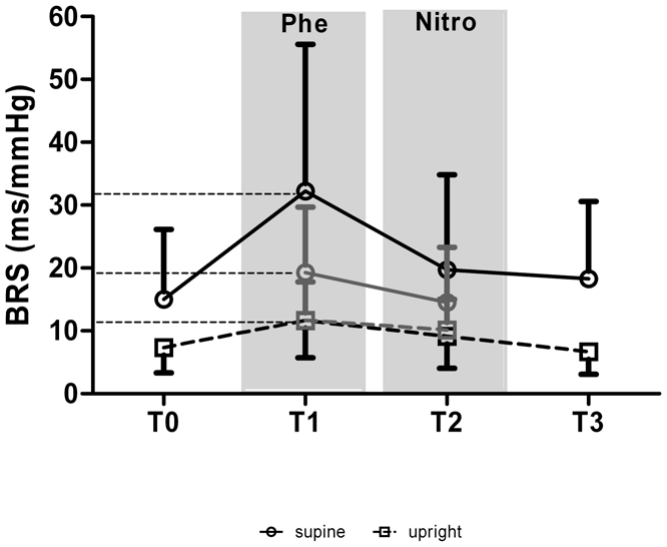
Time course of spectrally determined (black line) and of drug-induced (grey line) BRS during the course of the modified Oxford maneuver. T0, baseline; T1, phenylephrine; T2 nitroprusside; T3, recovery. Data are means ± SD.

The BRS estimates before, during and after the modified Oxford maneuver are shown in Figure 2. The spontaneous TRS-BRS using a *stationary* algorithm was higher in supine than in upright position (15.0±11.1 ms/mmHg vs. 7.3±4.0 ms/mmHg, p<0.001). The drug-induced Oxford-BRS during phenylephrine (Oxford-Phe) was higher than that during nitroprusside (Oxford-Nitro) (p<0.001). The Oxford-Phe and Oxford-Nitro were substantially correlated (supine: r = 0.81; standing: r = 0.71). Variance analysis of TRS-BRS during the modified Oxford maneuver (i.e. under *non-stationary* conditions) revealed an increase under phenylephrine (p<0.001) and a decrease under nitroprusside (p = 0.023) compared with the stationary BRS estimate before and after the maneuver. The phenylephrine-evoked change of BRS was more pronounced in the supine position than in the upright position (time x position interaction p = 0.001). All differences observed were independent of age and gender. Spontaneous TRS-BRS estimates measured before and after the Oxford maneuver did not differ.

**Figure 3:**
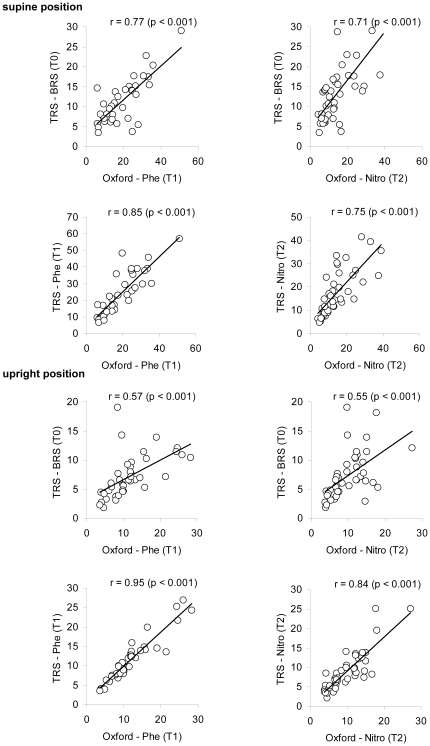
Correlation analysis of BRS estimates from the modified Oxford method and from trigonometric regressive spectral analysis (TRS). r, product-moment correlation coefficient; TRS-BRS, BRS determined by TRS under resting conditions; TRS-Phe, TRS-Nitro, BRS determined by TRS during application of phenylephrine (Phe) or nitroprusside (Nitro); Oxford-Phe, Oxford-Nitro, BRS determined by the modified Oxford maneuver using phenylephrine (Phe) or nitroprusside (Nitro).

There was a moderate to strong linear association between all BRS estimates as depicted in Figure 3. The non-stationary TRS-BRS during the Oxford maneuver was generally stronger associated with the drug-induced Oxford-BRS than the stationary TRS-BRS irrespective of the position. During standing, stationary TRS-BRS was only moderately correlated with Oxford-BRS.

### Test-retest reliability of BRS estimates

Test-retest reliability of BRS estimates determined by the modified Oxford maneuver (T1 vs. T2) was high in the lying position (r = 0.74) and moderate in the standing position (r = 0.63). Non-stationary BRS estimates determined by TRS during pharmacologically provocation (T1 vs. T2) were highly correlated in the lying position (r = 0.82) while the correlation in the standing position was somewhat lower (r = 0.65). The Pearson correlation coefficients for spontaneous BRS estimates determined by TRS during resting phases (T0 vs. T3) were 0.85 in the lying position and 0.69 in upright posture.

### Comparing methods of BRS estimation by the Bland-Altman method of differences

**Figure 4:**
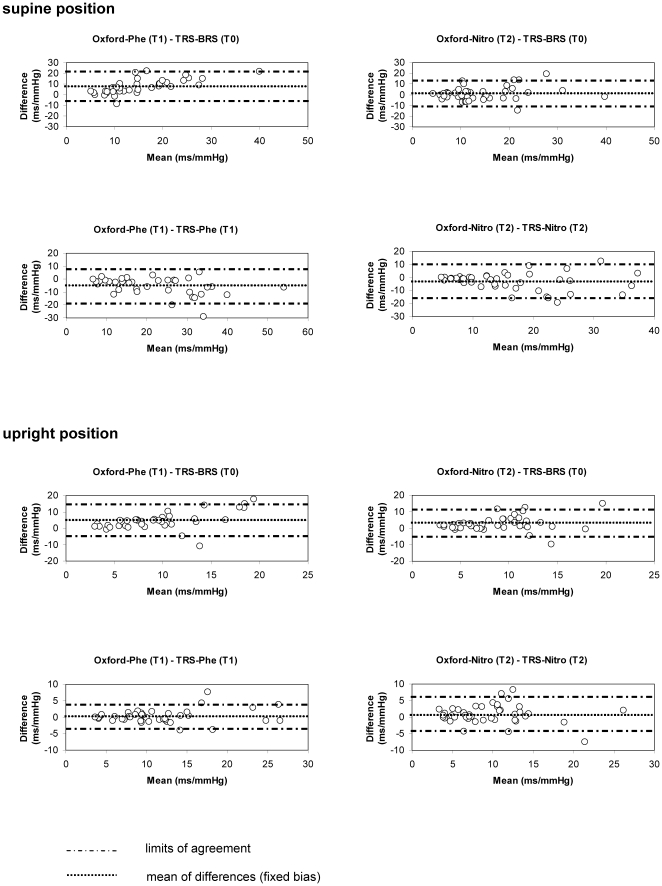
Bland-Altman Plots for comparison of BRS estimates obtained from the modified Oxford method and trigonometric regressive spectral analysis (TRS). TRS-BRS, BRS determined by TRS during resting conditions; TRS-Phe, TRS-Nitro, BRS determined by TRS during application of phenylephrine (Phe) or nitroprusside (Nitro); Oxford-Phe, Oxford-Nitro, BRS determined by the modified Oxford maneuver using phenylephrine (Phe) or nitroprusside (Nitro).

In Figure 4 the differences between BRS estimates determined by TRS and by the modified Oxford method (here as standard method) are plotted against the mean of the estimates from both methods.

#### Stationary TRS-BRS during resting conditions versus Oxford-BRS

In the lying position, stationary TRS-BRS underrated the BRS determined with phenylephrine during Oxford maneuver (Oxford-Phe) significantly by 7.9 ms/mmHg (95% CI -5.9 to 21.6 ms/mmHg). There was also a significant proportional bias due to an increase in scatter towards larger values leading to overestimation of limits of agreement of low BRS values and underestimation of limits of agreement of high BRS values (slope (b)  = 0.64 ms/mmHg, p<0.001). The stationary TRS-BRS estimates and Oxford-BRS estimates under nitroprusside (Oxford-Nitro) did not significantly deviate (95% CI −11.5 to 13.6 ms/mmHg). There was no proportional bias. In the upright position, stationary TRS-BRS underrated Oxford-Phe significantly by 4.5 ms/mmHg (95% CI −5.4 to 14.4 ms/mmHg) and Oxford-Nitro by 2.7 ms/mmHg (95% CI −5.7 to 11.1 ms/mmHg). There was a proportional bias between stationary TRS-BRS and Oxford-BRS under phenylephrine (b = 0.64, p<0.001).

#### Non-stationary TRS-BRS during pharmacological stimulation versus Oxford-BRS

TRS applied during phenylephrine and nitroprusside injection in supine position significantly overestimated Oxford-Phe by −5.2 ms/mmHg (95% CI −18.8 to 8.3 ms/mmHg) and Oxford-Nitro by −3.0 ms/mmHg (95% CI −16.1 to 10.0 ms/mmHg). There was a good agreement between the two methods in the upright position under phenylephrine and only a marginal underestimation of the BRS by the TRS method by 0.9 ms/mmHg (95% CI −4.6 to 6.4, p = 0.04) under nitroprusside. There was a proportional bias between non-stationary TRS-BRS and Oxford-BRS under phenylephrine in the supine position (b = −0.24, p = 0.02).

### Comparing methods of BRS estimation by ordinary least products regression

Applying OLP regression, TRS-BRS estimates did not systematically deviate from Oxford-BRS estimates indicating a good agreement between both methods (Table 1). There was a proportional bias when comparing stationary TRS-BRS with Oxford-Phe in both the supine and upright position and with Oxford- Nitro in upright position.

**Table 1:** Outcomes of comparison of BRS estimation methods by ordinary least products regression.

	supine				60° upright			
parameters	Oxford-Phe	Oxford-Nitro	Oxford-Phe	Oxford-Nitro	Oxford-Phe	Oxford-Nitro	Oxford-Phe	Oxford-Nitro
	vs.	vs.	vs.	vs.	vs.	vs.	vs.	vs.
	TRS-BRS	TRS-BRS	TRS-Phe	TRS-Nitro	TRS-BRS	TRS-BRS	TRS-Phe	TRS-Nitro
**a**' **(95% CI)**	−3.9	−2.7	−4.1	−4.4	−0.5	0.4	−0.5	0.9
	(−10.3–0.3)	(−13.8–2.4)	(−8.7–2.3)	(−16.0–2.0)	(−4.2–2.2)	(−2.5–2.6)	(−3.5–1.7)	(−1.9–3.1)
**b**' **(95% CI)**	2.0	1.3	0.9	1.2	1.9	1.5	1.2	1.1
	(1.7–2.7)	(0.9–2.3)	(0.7–1.2)	(0.7–2.0)	(1.4–2.5)	(1.1–2.0)	(0.9–1.5)	(0.8–1.4)
**proportional bias**	yes	no	no	no	yes	yes	no	no
**fixed bias**	no	no	no	no	no	no	no	no

a',b' - coefficients in ordinary least products regression model y  =  a' + b'x; a' – (y axis) intercept, b’ – slope; proportional bias, if 95% confidence interval (CI) for b' does not include 1; fixed bias, if 95% CI for a' does not include 0.

Abbreviations: TRS-BRS, spontaneous baroreflex sensitivity (BRS) determined by trigonometric spectral analysis (TRS); TRS-Phe/TRS-Nitro, BRS determination during phenylephrine/nitroprusside infusion using TRS; Oxford-Phe/Oxford-Nitro, BRS determination by phenylephrine/nitroprusside infusion (modified Oxford method).

## Discussion

Owing to its predictive power BRS determination is increasingly used as prognostic tool in cardiology and other medical specialties [19], [20], [21]. The current investigation primary aimed at comparison of the modified Oxford method as gold standard for BRS estimation with the spectrally determined BRS using the novel TRS algorithm. For the first time we also describe the dynamic modulation of BRS during the time course of a modified Oxford maneuver.

### Practicability of both methods

The modified Oxford maneuver was performed without complications. Methodological flaws of pharmacologic BRS estimation mainly reside in the manual selection of the raw data segment to be evaluated which lacks objectivity. As several authors have previously described the biphasic blood pressure response to phenylephrine infusion complicates an objective data evaluation [22], [23], [24], [25]. Considering that BRS estimates differ depending on the data segment selected for analysis, we strictly calculated drug-induced BRS only from the data of the first systolic blood pressure peak. This time-dependency of BRS calculation may be a result of the differences in the timing of reflex response mediated by sympathetic and parasympathetic systems. In fact, the initial heart rate response to vasopressors is parasympathetically mediated with a much more delayed latency of onset of sympathetic responses [26]. Direct and rapid pharmacologically effects of injected drugs may also potentially affect the shape of the sigmoidal baroreflex gain curve [9], [11]. Another methodical limitation was the selection of the phase shift between the blood pressure and the corresponding heart rate response. The physiological latency has been estimated with one or two heart beats [27] while the highest correlation between SBP and the corresponding RRI was determined with one-beat lag time [24]. Others again proposed that the SBP-RRI relationship might be dependent on the length of RRI [28], [29]. In the current study, we used the beat with the highest correlation between SBP and RRI considering delays of up to two beats. Owing to the transient nature of drug-induced changes unidirectional data sequences for linear regression comprise frequently not more than 10 heart beats which increases the statistical uncertainty of BRS estimation. The small number of coherent data pairs makes the modified Oxford method particularly prone to artifacts which, on the contrary, is minimized in the TRS method since it detects real physiological oscillations which are shifted beat by beat along a global data segment, thus providing a large number of statistically relevant TRS spectra [14]. The TRS method also only requires non-invasive assessment of pulse and blood pressure which can be done quickly and with minimal subject cooperation and stress. The computer-assisted spectral analysis of the raw data additionally guarantees a higher objectivity of TRS-BRS estimates.

### Comparison between stationary TRS-BRS and Oxford-BRS

Despite a close correlation of spectrally and pharmacologically determined BRS the Bland-Altman method of differences found a systematic deviation of the stationary TRS-BRS from the Oxford-BRS. The wide limits of agreement indicate that the deviation can be as large as the gain itself. The stationary TRS-BRS was consistently lower than the Oxford-BRS. This may reflect the different aspects of the cardiac baroreflex. The gain of the drug-induced BRS involves maximal recruitment of autonomic neurons under relatively strong activation while that of spontaneous BRS features minimal recruitment during cardiovascular steady-state. Previous method comparison studies, however, have yielded conflicting results [5], [7], [22], [25], [26], [30] what may be attributed to differences in algorithms used for calculation of spontaneous BRS estimates. While many authors perceive spontaneous BRS as valid alternative [6], [23], [25], [26] others argue that the extent of the observed systematic deviance render spontaneous estimates as inappropriate substitute [5], [7], [22], [30]. Considering that both methods involve different portions of the sigmoid BRS curve, spontaneous and drug-induced estimates can never be completely identical [31]. Furthermore, spontaneous variability of RRI and SBP may result from numerous interfering mechanisms including both feedback and feedforward aspects which naturally characterize a closed-loop control system. Opening the loop by drug-induced perturbation of the system enables a BRS estimation over a wide range of SBP and RRI leading to a more robust BRS estimate representing only feedback mechanisms [5]. Injections of vasoactive agents also represent stimuli of much higher intensity leading to far greater changes in SBP and RRI than typically observed under physiological conditions. This intensity effect could potentially lead to different responses between the methods [26]. It should also be noted that nitroprusside and phenylephrine elicit pharmacological alterations of baroreflex gain itself [11], [12], [32]. Another aspect to be considered is that the modified Oxford method cannot adequately account for the physiological variability in BRS owing to the limited amount of relevant data for BRS calculation. These naturally occurring short- and long-term variations in BRS are picked up by TRS owing to the use of single time windows which are shifted beat by beat for dynamic determination of spectral parameters. Thus, TRS may provide BRS estimates of higher reliability [21], [33]. In contrast to the Oxford method, TRS calculates BRS in the frequency domain matching the nonlinear characteristics of baroreflex gain better. It is generally assumed that the complex biological system of cardiovascular homeostasis is inadequately described by a simple linear relationship of isolated blood pressure and RRI sequences. Heart rate variability rather represents a stochastic process which seems adequately treated by TRS as it statistically predicts oscillations with maximal probability [14].

Using OLP regression, we did not find any systematic difference between the investigated methods. This discordance in output is rooted in inherent deficits of the Bland-Altman method of differences. First, tests for proportional and fixed bias are not independent. Ludbrook additionally argues that a deviation of the mean difference from zero does not necessarily reflect a fixed bias but may well be related to a mixture of proportional and fixed bias acting in the same direction. Second, with increasing mean values there is a proportional increase in the scatter of differences which inherently introduces fixed bias when the method of differences is used [34]. Since OLP regression allows for more accurate discrimination between fixed and proportional bias we regard the results of OLP more reliable.

### Comparison between non-stationary TRS-BRS and Oxford-BRS

This is the first study to assess oscillations of SBP and RRI during drug injection and to calculate non-stationary, dynamic TRS-BRS under brief and extreme pharmacological perturbations. We observed a time-dependent change in the gain characterised by a significant increase during phenylephrine and a significant decrease under nitroprusside. These drug-related variations in BRS are also evident in the Oxford-BRS. One explanation for this phenomenon originates in the sigmoidal shape of the SBP-RRI relationship where any initial pressure increase from an originally low resting point is answered by a relatively greater change in RRI due to approximation of the most linear portion [31]. Rudas et al. also refer to the fact that viscoelastic properties of the vascular wall favour propagation of stretch stimuli rather than stimuli induced by decreasing vascular tone [35]. Overall, we observed an approximation of TRS-BRS and Oxford-BRS under non-stationary (pharmacological) conditions evidenced by higher correlation coefficients, a smaller proportion of proportional and fixed bias and narrower limits of agreement. Estimates converged even more when standing possibly due to postural shifts in sympathovagal balance due to sympathetic activation [36]. Despite approximation between non-stationary TRS-BRS and Oxford-BRS, a complete agreement will not be achieved as data segments used for BRS calculation during pharmacological stimulation differ significantly in length between the methods.

### Study limitations and limitations of TRS method

The calculation of BRS over a small range of possible blood pressure changes by TRS poses a potential drawback of this method. On the other hand, it may provide gain around a physiological set point without artificial perturbation of the system. Lipman and colleagues criticised that spontaneous fluctuations might be at times immeasurably small, not covering the linear portion of the baroreflex response curve and involve an undistinguishable mix of feedback and feedforward aspects of the cardiovascular system. Still, there is evidence that the so called non-rhythmical fluctuations in heart rate are also relevant to cardiovascular control [37]. Thus spontaneous estimates may provide a broader picture of the baroreflex physiology than a drug-induced reduction of this complex control circuit to a feedback loop [12], [18], [38].

As low BRS values are demonstrably associated with a higher risk of cardiac mortality it is important to further investigate the prognostic ability of TRS in identifying persons at risk [2], [19]. Our study sample only comprised healthy individuals not allowing for the results to be conveyed to populations at risk or diseased populations. It also would have been interesting to compare TRS-BRS estimates with other risk parameters such as reduced left ventricular ejection fraction. However, those aspects warrants future investigations to built on our findings.

The underestimation of low BRS values from spontaneous oscillations by TRS may pose an important clinical limitation since individuals with BRS in the lower physiological range may be wrongly classified as at risk. Intriguingly, the EuroBaVar study did neither report an over- nor an underestimation of BRS by TRS with these estimates being in good agreement with other BRS estimates. In that study, TRS correctly identified individuals at high risk as reflected by low BRS estimates [13]. Since we did not include patients with pathological low BRS the estimation of values lower than<3 ms/mmHg where the risk of cardiac death is threefold [2] could not be evaluated in our study.

### Can the TRS method substitute the modified Oxford method?

Consistent with others [6], [26] our findings show that spontaneous TRS-BRS and Oxford-BRS are significantly correlated. They also display directionally similar changes under different conditions which according to Parati et al. [12] may reflect virtually superimposable baroreflex physiology. We, therefore, support the concept of complementarity where each method provides information about different aspects of baroreflex function. Since TRS allows for both stationary and non-stationary analysis it is able to evaluate the full spectrum of possible baroreflex gains with high reliability. Given the invasiveness of the modified Oxford method, its pharmacological inference with baroreflex function and its limited reproducibility we perceive the TRS method as preferable for clinical use. This particularly accounts for children or diseased patients in whom injection of vasoactive substances is often contraindicated. The clinical applicability of the TRS method is substantiated by own investigations in children and Parkinson patients [15], [18], [21].

### Perspectives

The TRS method is a non-invasive and reliable tool of BRS estimation applicable under both steady state and variable cardiovascular conditions. Future studies should aim at establishing age- and gender-specific normative values which are essential for cardiovascular risk stratification. As mentioned above, future investigations should aim at verification of TRS as risk stratification and prognostic tool in populations at risk as well as in diseased. As there is a constant need for validated biomarkers of therapeutic efficacy the performance of TRS as non-invasive tool for the dynamic evaluation could be investigated in therapeutic interventions.

## Methods

### Participants and Ethics

Forty-five healthy volunteers were investigated in a cross-sectional study at the Department of Neurology of the University Hospital Dresden, Germany. Volunteers were excluded when they had a history of diabetes mellitus, arterial hypertension, cardiovascular disease and renal disease or when on regular medication directly affecting autonomic or cardiovascular function. Resting blood pressures and electrocardiogram (ECG) were evaluated for any pathological signs. All participants received detailed verbal and written information about the study objectives and procedure, and gave written informed consent. The study was approved by the Ethics Committee of the Faculty of Medicine of the Dresden University of Technology and the study procedure performed with the Declaration of Helsinki.

### Laboratory Autonomic Assessment

All recordings were performed on a tilt table in a temperature and humidity controlled specialized autonomic laboratory. Food intake, use of medication and consumption of caffeine and nicotine had to be ceased at least 3 h before the examination. After insertion of an indwelling catheter continuous cardiovascular monitoring was performed using the SUEMPATHY device (Suess Medizin-Technik, Aue, Germany) including the non-invasive blood pressure monitoring CBM7000 device (Colin Instruments, Houston, Texas, USA). Respiratory frequency and heart rate were simultaneously recorded using a piezo-resistive belt and a 3-channel ECG (sampling frequency 512 Hz, digitalized 12 bit). After instrumentation, calibration and a test measurement data acquisition commenced with the participant in the supine position (Table 2). According to the protocol each drug administration phase of 2 min was preceded by 2 min of steady state recordings and followed by 5 min of recovery recordings to allow for SBP and RRI to return to baseline. This protocol was performed twice in supine position and in 60° upright position respectively using the head-up tilt-table test (HUT). The purpose of the HUT was to additionally evaluate both methods under the effect of orthostasis. For HUT, patients were tilted up to a 60° upright position within 15 s after being in a resting supine position for a minimum of 10 min.

**Table 2:** Experimental protocol.

phase	process (patient in supine position)	duration (min)
**1.**	instrumentation, calibration, test measurement	20
**2.**	recovery	5
**3.**	steady state	2
**4.**	bolus administration of phenylephrine and nitroprusside	2
**4.**	recovery	5
**5.**	bolus administration of phenylephrine and nitroprusside	2
**6.**	recovery	5
**7a.**	steady state	6
**7b.**	HUT	5
**7c.**	steady state	2
**7d.**	administration of phenylephrine and nitroprusside	2
**7e.**	recovery after backward tilt	8
**8.**	repetition of 7.	23

### Modified Oxford Maneuver

**Figure 5:**
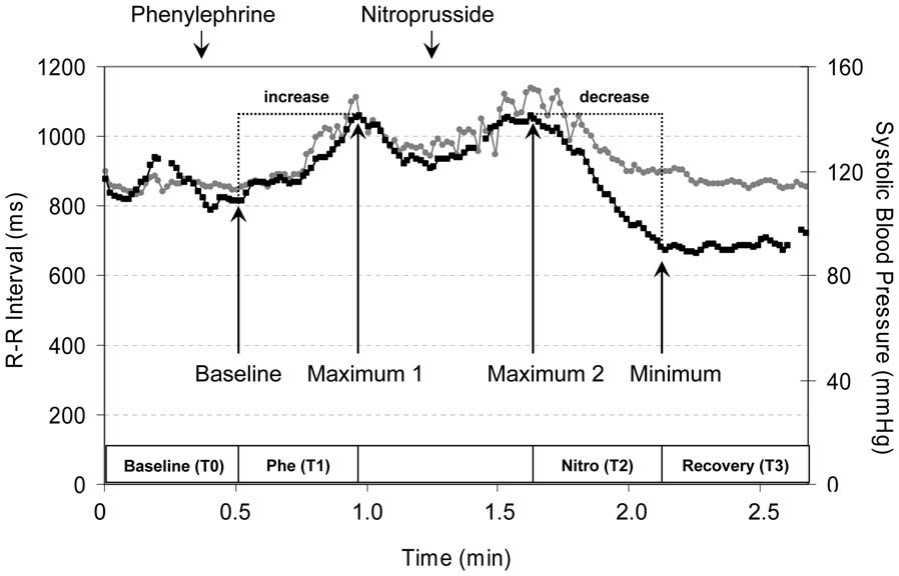
Exemplified time course of systolic blood pressure (black line) and R-R interval (grey line) during the modified Oxford maneuver.

The time course of the modified Oxford maneuver is shown in Figure 5. To evoke significant blood pressure alteration an intravenous bolus of 150 µg phenylephrine hydrochloride and 100 µg sodium nitroprusside was consecutively administered with an interval of 60 s between the injections [7]. The dose of the repeated injection was individually adjusted according to the amplitude of the blood pressure alteration, which had to be at least 15 mmHg. Ectopic beats were filtered from raw recordings.

BRS was calculated by means of linear regression where each RRI was plotted as a function of SBP. The analysis interval was defined as the data between the beginning and the end of the first increase in SBP after the phenylephrine injection (Oxford-Phe, T1) and the beginning and the end of the decrease in SBP after nitroprusside injection (Oxford-Nitro, T2). Each systolic pressure peak was coupled to the RRI with the highest correlation lagging between 0 and 2 heart beats. Only data-pairs with r>0.7 and with a systolic pressure increment of at least 15 mmHg were used for calculation of Oxford-BRS. The two consecutive tests were averaged within each subject in the respective position [7], [22].

### Trigonometric regressive spectral analysis

**Figure 6:**
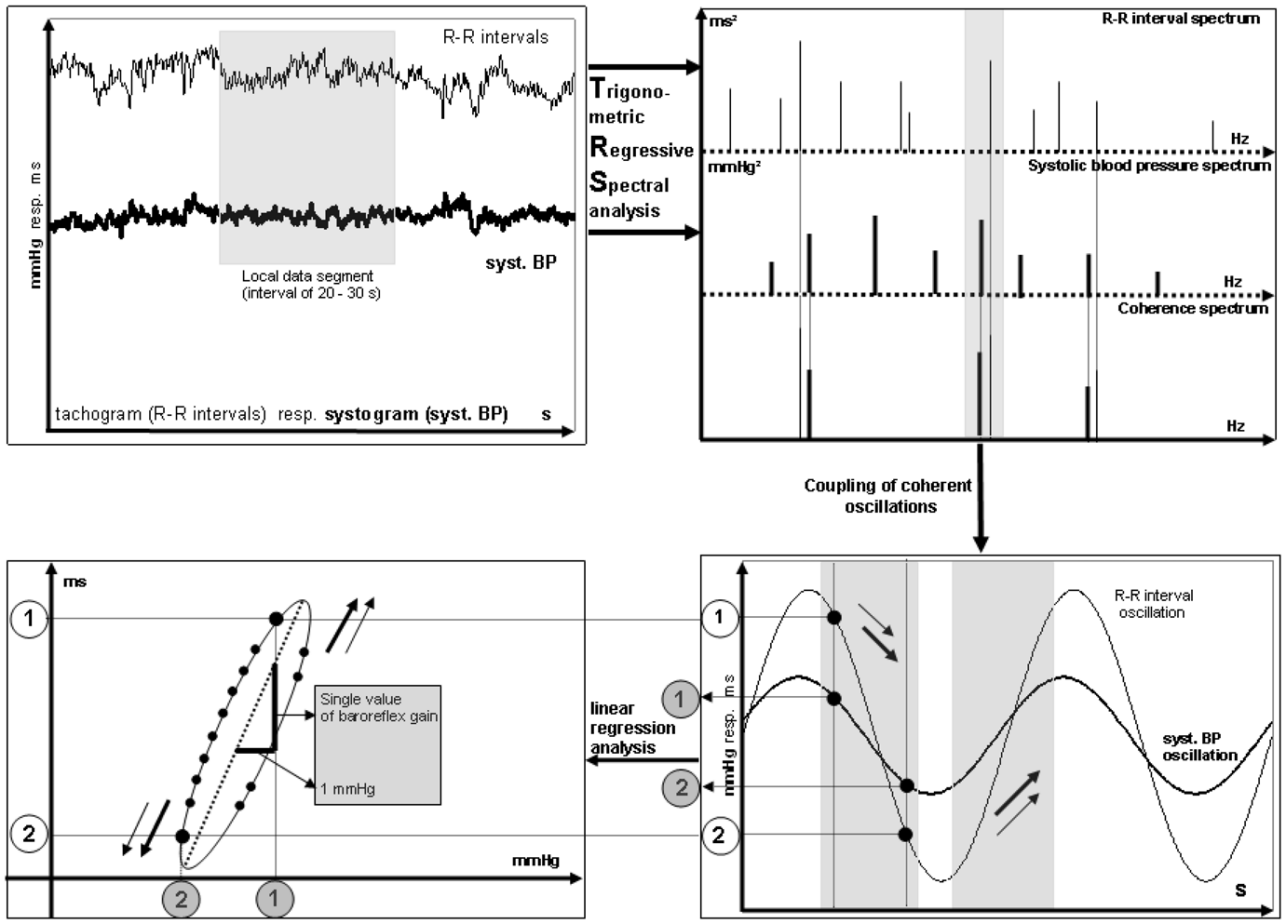
Illustration of BRS estimation process. Spontaneous oscillations (top left) are replaced by theoretical TRS oscillations (top right). Calculation of BRS as slope of the regression line (bottom left) originating from coherent oscillation pairs (bottom right).

TRS analysis (ANS Consult, Freital, Germany) was applied to spontaneous oscillations of SBP and RRI before (T0) and after (T3) each modified Oxford maneuver. Nonartifactual stationary global data segments of 1.5–2 min were analyzed using single time windows of 25 s which were successively shifted by 5 beats for temporal determination of frequency and amplitude. Analogous to the modified Oxford maneuver, TRS oscillations are determined by regression analysis differing only in the described relationship which is of trigonometric and not linear origin. Each predicted TRS oscillation reduces the total variance of the original process. All oscillations are detected under the condition that the deviance from the sinusoidal regression line (y(t_i_) – Reg(t_i_))^2^ is minimal where y(t_i_) being the original RRI or SBP and Reg(t_i_)  = A x sin (ωt_i_ + ϕ_i_) being a trigonometric function of the parameter A (amplitude), ω (frequency) and ϕ (phase shift). The BRS (TRS-BRS) was calculated from coherent pairs of the detected oscillations of RRI and SBP (cross correlation coefficient >0.7) (Figure 6) [15], [16], [17]. The estimation of a global TRS-BRS index was based on the weighted mean of all 300–500 individual BRS values derived from a data segment according to a variance ratio: (variance reduction of RRI/variance reduction of SBP)^2^.

For determination of baroreflex sensitivity using the TRS technique (TRS-BRS) during the application of phenylephrine (T1) and nitroprusside (T2) a shorter local data segment of 20 s was used which was shifted beat by beat in non-stationary mode. This mode of analysis enabled us to calculate BRS for the first time during the drug injection phase so that we reached a continuous BRS before, after (stationary TRS-BRS) and during (non-stationary TRS-BRS) the Oxford maneuver.

The EuroBavar Study demonstrated a good agreement of BRS calculated by TRS with that obtained by other non-invasive techniques [13]. A summary of the algorithm can be found on www.ans-consult.de and on the website of the European Working Group on Blood Pressure and Heart rate Variability (http://www.cbi.dongnocchi.it/glossary/eurobavar.html).

### Statistical methods

The SPSS software package version 16.0 for Windows (SPSS Inc., Chicago, IL, USA) was used for all statistical computations. Data are presented as mean ± SD unless stated otherwise. Distribution of data was determined by Kolmogorov-Smirnov test. Univariate repeated measures ANOVA with age and gender as covariates was applied to test for differences in the time course of spectrally determined TRS-BRS. For differences between independent variables SIDAK correction was applied. To compare the two methods of BRS determination we performed Pearson product-moment correlation, the Bland-Altman method of differences and the ordinary least products (OLP) regression. According to the Bland-Altman method, the differences between the pairs of measurements (Oxford-BRS – TRS-BRS) on the vertical axis were plotted against the mean of each pair ((Oxford-BRS + TRS-BRS)/2) on the horizontal axis [34], [39]. For determination of fixed and proportional bias we calculated the mean of the difference (*d*) and the slope of the regression line fitted to the plot. If neither fixed nor proportional bias is present between the two methods, then *d* and the slope of the regression line is zero [34]. We also calculated the 95% confidence intervals of the differences (the so called limits of agreement [39]), given by *d* plus or minus twice the standard deviation of *d*. T-test for independent samples was applied to test whether *d* or the slope of the least squares regression line calculated as Pearson correlation coefficient r significantly deviate from zero. P values<0.05 were considered statistically significant.

In a second step we applied OLP regression because of the following reasons: In biological systems there is usually an upward trend of differences with increasing mean as shown by r>0. This upward trend introduces bias into the estimates of slope which may in turn lead to over- or underestimation of limits of agreement. Furthermore, if there is a proportional bias, then *d* will almost inevitably deviate from zero. Thus, fixed bias may be over- or underdiagnosed. Last but not least, we expected random errors within both methods which are taken into account by the OLP method [5], [22], [34], [40]. The coefficient for the slope and the intercept of the OLP regression y  =  a' + b'x were calculated from the ordinary least squares equations E(y)  =  a + bx und E(x)  =  a + by according to the formula b'  =  sqrt((b_y,x_)(1/b_x,y_)) and a'  =  E(y) - b' E(x). E(x) and E(y) are the estimated values of the mean of x and y. The 95% confidence interval (CI) for a' and b' was calculated by bootstrapping. A proportional error exists when the 95% CI of b' includes 1. A fixed bias exists when the 95% CI of a' includes 0 [34].
